# State-of-Charge-Dependent Deformation and Electrochemical Evolution in Sodium-Ion Batteries Under Mechanical Compression

**DOI:** 10.3390/molecules31101652

**Published:** 2026-05-14

**Authors:** Shudong He, Xiong Shu, Yulong Dai, Wenxian Yang

**Affiliations:** 1 School of Intelligent Manufacturing and Mechanical Engineering, Hunan Institute of Technology, Hengyang 421002, China; 2 Hunan Provincial Key Laboratory of Vehicle Power and Transmission System, Hunan Institute of Engineering, Xiangtan 411104, China; 3 School of Computing and Engineering, University of Huddersfield, Huddersfield HD1 3DH, UK

**Keywords:** morphological, EIS, state of health, sodium-ion batteries

## Abstract

Sodium-ion batteries (SIBs) are emerging as attractive electrochemical energy-storage systems owing to the natural abundance and low cost of sodium resources. However, their structural integrity and electrochemical stability under mechanical abuse remain insufficiently understood, particularly from the perspective of coupled morphological and transport responses in porous electrode assemblies. In this work, the material deformation behavior and electrochemical evolution of SIBs under compressional loading are systematically investigated, with particular attention to the roles of state of charge (SOC), electrode microstructure, and separator integrity. Electrochemical impedance analysis reveals that the ohmic response is mainly dominated by the extent of compressional deformation, whereas interfacial and diffusion-related resistances are jointly regulated by deformation and SOC. In particular, elevated SOC significantly intensifies the increase in diffusion impedance during compression, indicating a strong coupling between sodium-storage state and mass-transport deterioration. Moreover, cells at higher SOCs exhibit accelerated open-circuit voltage decay during extrusion, suggesting enhanced internal stress accumulation and aggravated instability of the electrode/electrolyte interface. Post-mortem morphological characterization demonstrates substantial particle fracture, pore collapse, and crack propagation in both cathode and anode materials, accompanied by severe shrinkage and partial destruction of the separator microporous network. These results establish a direct correlation between compressional deformation, microstructural damage, and electrochemical degradation in SIBs, and provide useful insights for the design of mechanically resilient electrode architectures, separator materials, and safety-oriented diagnostic strategies for next-generation sodium-ion energy-storage devices.

## 1. Introduction

With the global transition of the energy structure toward low-carbon and sustainable development, efficient, safe, and cost-effective electrochemical energy storage technologies have become an essential foundation for supporting the large-scale utilization of renewable energy. Among the various energy storage technologies, lithium-ion batteries have been widely applied in electric vehicles and energy storage systems due to their high energy density, long cycle life, and well-established industrial supply chain [1,2,3,4]. However, the limited reserves of lithium resources and their uneven global distribution have led to continuously increasing resource acquisition costs [5], which to some extent restricts the further large-scale deployment of lithium-ion batteries in grid-level energy storage applications. Therefore, the development of new energy storage systems with abundant resources and lower costs has become an important research direction in the field of electrochemical energy storage.

Among the various candidate technologies, sodium-ion batteries have attracted extensive attention because of their abundant resources, low cost, and working principles similar to those of lithium-ion batteries [6,7,8]. Sodium is far more abundant in the Earth’s crust than lithium and is more evenly distributed worldwide, resulting in a more stable supply of raw materials. In addition, sodium-ion batteries can typically employ lower-concentration electrolyte systems and more inexpensive current collector materials, thereby further reducing manufacturing costs. In recent years, with the continuous development of key electrode materials, including layered oxides, Prussian blue analogs, and hard carbon anodes [9,10,11,12], sodium-ion batteries have achieved significant progress in terms of energy density, rate capability, and cycling stability [13,14]. As a result, they have demonstrated considerable application potential in fields such as grid energy storage, low-speed electric vehicles, and distributed energy systems.

Although sodium-ion batteries have achieved significant progress in terms of electrochemical performance, their safety and reliability under complex engineering environments remain critical factors limiting their further practical applications [15,16]. In practical applications, battery systems may be subjected to various types of mechanical loads, such as vehicle collisions, transportation-induced vibrations, drop impacts, and structural compression in energy storage systems [17]. These external mechanical loads may cause deformation or even structural damage to the battery, thereby triggering a series of coupled failure processes, including electrode material fracture, separator penetration, electrolyte leakage, and internal short circuits, which may ultimately result in localized overheating or even thermal runaway [18,19]. Therefore, investigating the failure mechanisms of batteries under mechanical deformation conditions is of great significance for improving the safety and reliability of energy storage systems.

In response to the failure behavior of batteries under mechanical loading conditions, extensive research efforts have been carried out in recent years, particularly in the field of lithium-ion batteries. For example, Wang et al. [20] reviewed the aging mechanisms of lithium-ion batteries and summarized recent advances in diagnostic methods for battery degradation in automotive applications. Gao et al. [21] investigated the mechanical response and structural deformation behavior of battery modules under external mechanical loading through combined experimental and simulation approaches. Bai et al. [22] investigated the influence of mechanical extrusion on the thermal runaway behavior of lithium-ion batteries under heating conditions and demonstrated that mechanical deformation significantly accelerates battery thermal instability. Zhu et al. [23] experimentally analyzed the internal short-circuit formation and failure mechanisms of lithium-ion pouch cells under mechanical indentation abuse. Xu et al. [24] studied the mechanical behavior and thermal runaway characteristics of automotive lithium-ion batteries under mechanical loading conditions. Xi et al. [25] investigated the mechanical response and structural deformation behavior of battery modules under external mechanical loading through experimental and simulation analysis. Chen et al. [26] revealed the transition mechanism from benign to catastrophic failure during external short-circuit events in lithium-ion batteries. Qu et al. [27] investigated the influence of cycling aging on the mechanical abuse behavior of lithium-ion batteries under extreme indentation conditions. Shu et al. [28] experimentally analyzed the internal short-circuit formation and failure mechanisms of lithium-ion pouch cells under mechanical indentation abuse conditions. Xiong et al. [29] studied the failure mechanisms and characteristic evolution of lithium-ion batteries under combined temperature conditions and extrusion deformation. At the same time, multiphysics coupling models have been widely employed to analyze the structural response of batteries under mechanical impact or compression conditions and their corresponding influence on electrochemical behavior. These studies provide an important theoretical foundation for understanding the safety issues of lithium-ion batteries under mechanical stress.

However, compared with lithium-ion batteries, research on the failure behavior of sodium-ion batteries under mechanical deformation conditions remains relatively limited. Existing studies mainly focus on the material level; Xing et al. [30] developed a self-assembled naphthalene diimide-derived nanostructured cathode to improve the electrochemical performance of sodium–organic batteries. Mathiyalagan et al. [31] reviewed the recent progress, challenges, and development strategies of layered transition-metal oxide cathodes for high-energy sodium-ion batteries. Mathiyalagan et al. [32] improved the electrochemical performance of sodium-ion batteries by optimizing the layered O3-Na_0.95_CrO_2_ cathode material. Feng et al. [33] enhanced the electrochemical performance of sodium-ion batteries through multi-elemental doping engineering of P2-type layered cathode materials. Chen et al. [34] proposed a three-dimensional hierarchical metallic scaffold with enhanced sodiophilicity to enable stable sodium metal anodes for high-performance sodium batteries. However, systematic studies on the response mechanisms of the entire battery structure under mechanical loading and their influence on electrochemical performance remain relatively limited. In fact, due to the larger ionic radius of sodium ions and the structural differences in electrode materials, sodium-ion batteries may exhibit structural evolution behaviors under mechanical stress that differ from those of lithium-ion batteries. For example, under external compression, electrode particles may fracture or undergo rearrangement, leading to an increase in interfacial contact resistance. The separator may rupture in regions with localized stress concentration, thereby triggering internal short circuits. Meanwhile, mechanical damage may also alter the ion transport pathways within the electrodes, which subsequently affects the electrochemical performance of the battery. In addition, complex coupling interactions usually exist among mechanical stress, electrochemical reactions, and thermal effects, causing the battery failure process to exhibit pronounced multiphysics coupling characteristics. Therefore, systematically investigating the behavior of sodium-ion batteries under mechanical compression from a mechanical–electrochemical–thermal coupling perspective is of great significance for achieving a deeper understanding of their failure mechanisms.

Although some studies have begun to focus on the safety issues of sodium-ion batteries in recent years, most existing research has mainly concentrated on thermal stability and electrochemical performance analyses. In contrast, studies concerning structural damage induced by mechanical deformation and its influence on battery performance remain insufficient. Particularly in practical engineering applications, batteries are often subjected to complex mechanical environments, and the evolution of internal structures and the corresponding changes in electrochemical behavior have not yet been systematically investigated through experimental approaches. Therefore, conducting research on the multiphysics coupling behavior of sodium-ion batteries under mechanical compression conditions is of great importance for filling the current research gap and promoting their practical engineering applications.

Based on the above research background, this study takes cylindrical sodium-ion batteries as the research object and systematically investigates their structural evolution, electrochemical performance variation, and potential failure mechanisms under external compression loads. By constructing a mechanical loading experimental platform, the voltage response, temperature variation, and structural damage characteristics of the batteries under different compression deformation conditions are experimentally analyzed. Furthermore, combined with multiphysics coupling theory, the relationship between internal structural damage and performance degradation of batteries under compression conditions is explored in depth. The results of this study are expected to reveal the failure mechanisms of sodium-ion batteries under mechanical stress and provide a theoretical basis for battery structural design and safety evaluation.

Overall, the main contributions of this work can be summarized as follows:

1. A mechanical compression experimental platform for sodium-ion batteries was constructed to enable systematic characterization of the battery structural deformation process.
2. The variation characteristics of the electrochemical performance of batteries under different compression deformation conditions were systematically analyzed.
3. The coupling relationship between internal structural damage and performance degradation of batteries under mechanical deformation was revealed.
4. The findings provide theoretical references for the safety-oriented design and reliability evaluation of sodium-ion batteries in practical engineering applications.

This study contributes to a deeper understanding of the behavioral characteristics of sodium-ion batteries under complex mechanical environments and provides important scientific insights for the safety design and optimization of emerging energy storage systems.

## 2. Methods and Test Rig

### 2.1. Testing Rig

**Figure 1:**
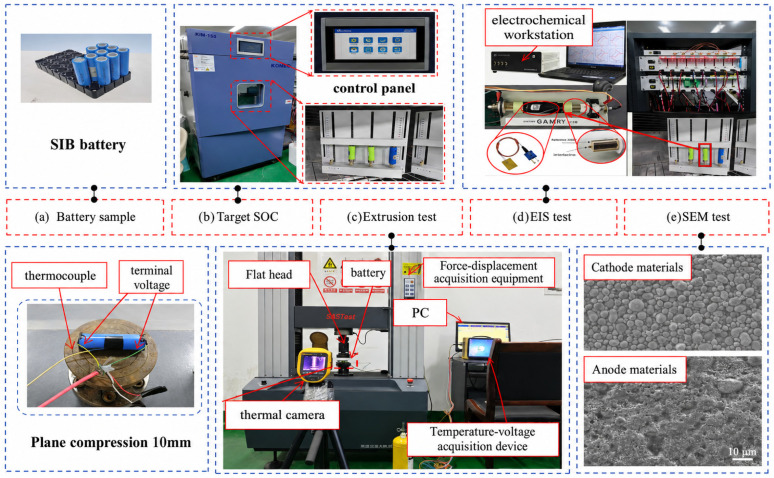
Experimental test platform.

To investigate the failure mechanism and its evolution of SIBs during compression deformation, we selected SIBs produced by a company as the research object for this study. The specific parameters of the batteries are shown in Table 1. A professional test rig was designed for experimental use with batteries, as illustrated in Figure 1. It is important to note that during this experimental test, all test items were conducted at an ambient temperature of 25 ± 2 °C, unless otherwise specified. The ambient temperature of 25 ± 2 °C represents standard room temperature conditions commonly used in mechanical abuse protocols, such as those in SAE J2464 [35] and UL (underwriters Laboratories) 2580 [36], to isolate deformation effects without confounding thermal variables, as batteries in operational environments often function near this range before extreme events. In addition, due to the professionalism of the test content, we will discuss the specific procedures of each test section separately.

As shown in Figure 1, the specific designed test platform consists of a temperature chamber, multifunctional squeeze tester, data monitoring software, charging and discharging equipment, and electrochemical workstation. After selecting the SIBs samples to be tested, the samples were first placed in an temperature chamber and charged to the target SOC, and then the SIBs were subjected to extrusion testing using multifunctional extrusion test equipment, the impedance spectra of the batteries were tested at different extrusion displacements using electrochemical workstations, the thermal diffusion of the batteries during extrusion were monitored by a thermal imaging camera, and the OCV, surface temperature, and stress information during the testing process were collected by the data acquisition equipment in real time. The temperature chamber is a key component of the entire test platform and is designed to simulate the ambient temperature conditions of the battery in real applications, and the performance of the battery at different temperatures can be investigated by precisely controlling the temperature. The electrochemical workstation is capable of performing EIS, a technique employed to analyze the impedance characteristics of the electrochemical system. The impedance of the electrode system is measured by applying alternating current (AC) signals of different frequencies, and the resulting impedance spectra are obtained. These provide information about the charge transfer resistance, ion diffusion, electrolyte and conductivity under different states. Meanwhile, the morphological characteristics and X-ray diffraction (XRD) patterns are examined by scanning electron microscopy (SEM) and X-ray diffraction, respectively. The model of the equipment used for SEM testing of sodium-ion batteries in the testing process is (JCM-7000 benchtop scanning electron microscope (JEOL Ltd., Akishima, Tokyo, Japan); the equipment used for EIS testing is CHI700E electrochemical workstation (Shanghai Chenhua Instrument Co., Ltd., Shanghai, China), and the equipment used for XRD testing is SmartLab SE multipurpose X-ray diffractometer (Rigaku Corporation, Akishima, Tokyo, Japan). To clarify, all were conducted under controlled ambient conditions at a constant room temperature of 25 ± 2 °C, maintained using the laboratory’s environmental control system to minimize thermal variations that could influence battery behavior. However, the battery’s SOC is always measured in a constant temperature chamber. The key parameters for the Charging/Discharging equipment and Electrochemical workstation are shown in Table 2.

### 2.2. Consistency Testing

To ensure the validity and reliability of the study results, consistency testing was conducted on the selected battery samples prior to the start of the experiments. These tests assessed key parameters such as initial capacity, internal AC resistance, and nominal voltage distribution to confirm that the selected batteries exhibited good consistency and met the research requirements. By randomly selecting 20 batteries for testing, the test parameters of the selected batteries were all within the specified error range given by the manufacturer (the allowable error range for the battery parameters provided by the manufacturer is shown in Table 1). This approach not only evaluated the output characteristics and EIS profiles of the SIBs in their initial state but also contributed to the development of more precise testing procedures and equipment parameters, ultimately enhancing the reliability of the testing process.

### 2.3. Force-Displacement-Temperature-OCV Characteristics, Thermal Diffusion Analysis, and EIS Measurements

In practical applications, SIBs are susceptible to external forces such as impact or compression, which can lead to mechanical and electrochemical failure. To investigate the coupling between mechanical deformation, thermal response, and electrochemical performance, we monitored stress, displacement, temperature, and voltage under well-defined compression conditions.

During the test, a multi-channel data acquisition system simultaneously collected data from pressure sensors, displacement sensors, temperature sensors, and voltage monitoring modules, ensuring real-time recording of all parameters. Compression was applied at a controlled loading rate of 1 mm/min, with a maximum displacement of 8 mm. Displacement sensors recorded instantaneous radial deformation at each step, while a thermal imager captured the global and localized temperature evolution. Thermocouples were affixed using thermally conductive adhesive, and temperature acquisition was synchronized with mechanical loading through the data acquisition module. Voltage monitoring captured OCV fluctuations to assess the effect of mechanical stress on electrochemical reactions. Electrochemical impedance spectroscopy (EIS) measurements were conducted prior to each compression test to quantify the internal resistance and electrode kinetics. EIS was performed over a frequency range of 100 kHz to 0.01 Hz with a 10 mV AC perturbation at the selected SOC, after a 30 min rest period to allow equilibration. This provided baseline impedance data for comparison with post-compression performance and enabled correlation of mechanical stress and diffusion-induced degradation with changes in charge transfer resistance and ionic diffusion pathways.

The SOC was defined quantitatively based on nominal capacity and verified against the measured OCV. SOC = 0% corresponds to the fully discharged state, SOC = 50% represents the nominal charge state, and SOC = 90% reflects the near-fully charged state. These SOC values were chosen according to international battery abuse testing standards (UL 2580, SAE J2464) to capture varying energy levels and failure risks while minimizing hazards. SOC = 100% was excluded due to safety concerns identified during preliminary testing, where full-charge cells exhibited a high risk of thermal runaway under compression.

Data on displacement, temperature, and OCV changes under different compression conditions were analyzed to investigate the relationship between mechanical deformation, heat generation, and electrochemical response. By comparing temperature rises and OCV fluctuations across different SOCs and applied stresses, we identified signs of internal material damage, thermal runaway, and failure mechanisms. This comprehensive approach, combining mechanical, thermal, and electrochemical measurements with EIS characterization, ensures reproducibility and allows quantitative interpretation of the coupled degradation phenomena in SIBs.

### 2.4. Morphological and Phase Characteristics

**Figure 2:**
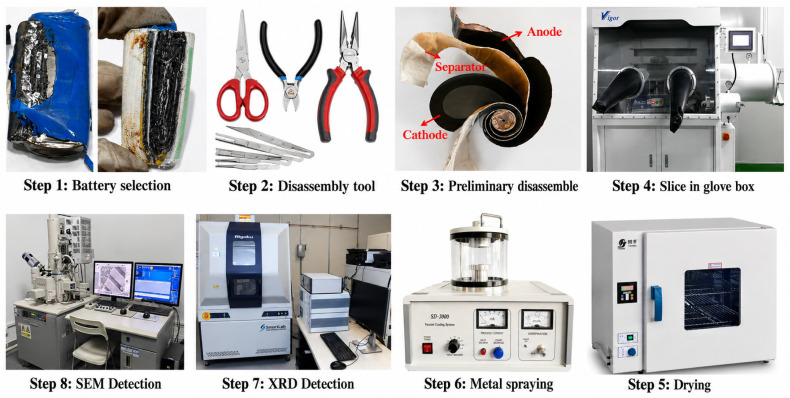
Procedures of morphological and phase characteristics test.

Scanning electron microscopy (SEM) is a commonly used tool for observing the microscopic surface structure of materials, offering high-resolution imaging of electrode and separator materials in SIB. Post-compression, the electrode and separator structures may sustain damage, significantly altering the battery’s conductivity, ion migration paths, and overall performance. SEM analysis was employed to observe microstructural changes induced by compression, including microcracks and particle fractures, pore structure and channel variations, byproduct deposition, and surface morphology changes. X-ray diffraction, an essential technique for analyzing the crystal structure of materials, provided information about the crystal phases of the electrode materials in compressed batteries. XRD analysis revealed the impact of mechanical stress on crystal structure and phase transitions, as well as potential byproducts resulting from electrolyte–electrode reactions during compression. In this study, the detailed testing procedures are illustrated in Figure 2.

For morphological and phase characterization, the extruded SIBs were first disassembled and spread out flat. The slices are then processed in a glove box with special tools. To ensure the accuracy of the results, 3 samples are cut as an alternative for each slice, and then they are dried in a baking oven in preparation for testing. In the SEM test, the conductivity of the sample surface should be decided according to whether or not to spray gold treatment.

## 3. Results and Discussion

### 3.1. Structural Damage Analysis

**Figure 3:**
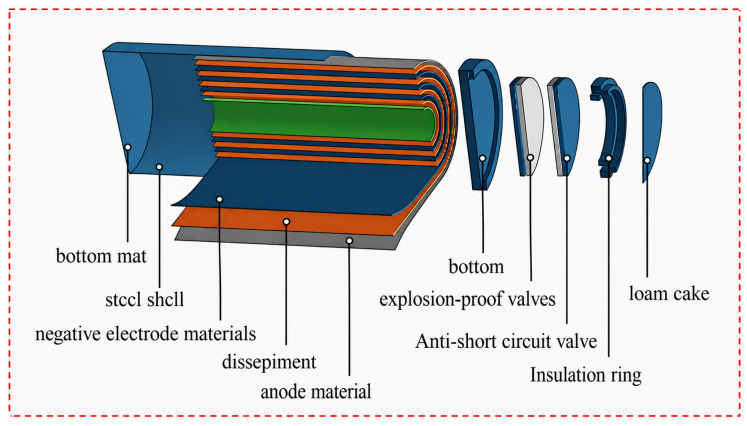
Structure of the cylindrical SIBs.

**Figure 4:**
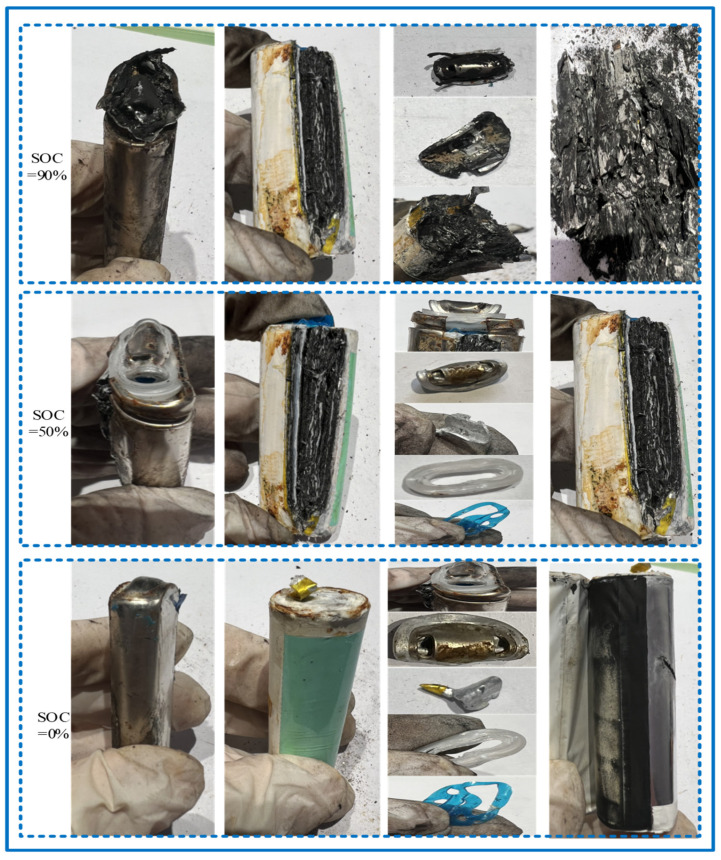
Structural damage in SIBs after compression.

The cylindrical sodium-ion batteries (SIBs), similar to the widely used 18,650 lithium-ion batteries (LIBs), consist of similar components, including the cathode and anode electrode sheets, separators, electrolyte, winding structures, top caps, and sealing rings, which are all essential for the battery’s function and structure, as shown in Figure 3. However, the materials and characteristics of the electrodes and electrolyte in SIBs differ significantly from those in LIBs. For example, the anode in SIBs is typically made of hard carbon or other sodium-friendly materials, whereas LIBs often use graphite or silicon-based anodes. Additionally, the electrolyte in SIBs is usually sodium salt-based, compared to lithium salts used in LIBs. These differences influence the performance and stability of the battery. Compression testing revealed significant damage to the tabs, winding structures, top caps, and sealing rings, and the results are shown in Figure 4.

From Figure 4, we can see that the cylindrical SIBs exhibited significant deformation under compression at different SOC levels. Similar to the behavior of 18,650 LIBs under compression [29], electrolyte leakage occurred in the SIBs, and cracks or weld failures appeared at the connections between tabs and electrode sheets. The top cap was dented and cracked, while both the sealing rings and gaskets experienced compressive deformation, highlighting structural weaknesses in both types of batteries under mechanical stress. A comparison of compression effects at different SOC levels revealed that SIBs with higher SOCs suffered more severe structural deformation than those with lower SOCs, which is also consistent with observations in LIBs. Specifically, samples with a SOC of 90% emitted slight explosive sounds during compression, and their internal winding structures exhibited pulverizing fractures. Additionally, samples with a SOC of 90% showed distinct burn marks on the sealing rings, which were absent in those with SOCs of 0% and 50%. This indicates that SIBs are vulnerable to thermal runaway under high SOC conditions, but the SIBs experienced even more severe structural damage due to the difference in ionic chemistry. The similar research in [34] shows that the 3D hierarchical metallic scaffolds for sodium metal anodes with enhanced sodiophilicity provides a complementary approach to improving anode stability.

Apart from that, the higher risk of thermal runaway in SIBs can be attributed to the larger ionic radius of sodium, which may result in less efficient ion diffusion and higher internal resistance, exacerbating overheating and damage under high charge conditions.

### 3.2. EIS Profile Feature Under Different Deformation

**Figure 5:**
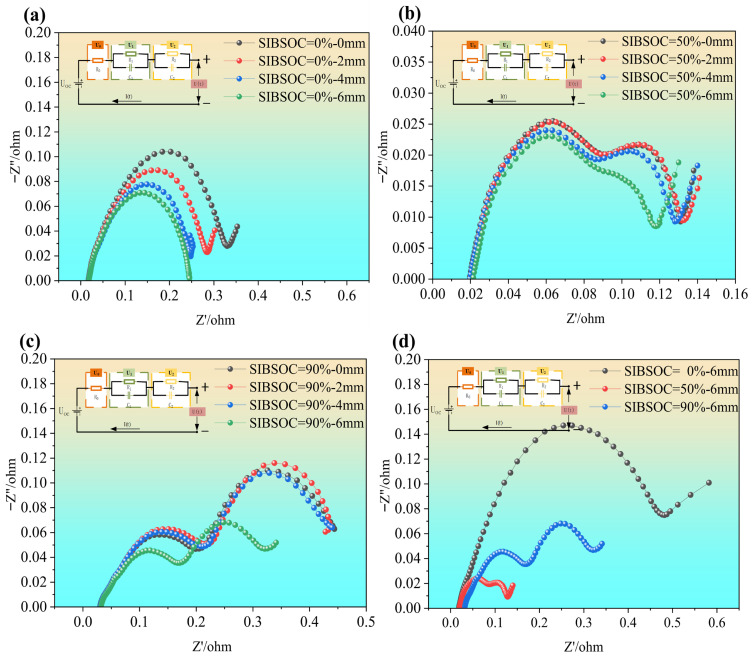
EIS profile of SIBs in different states: (**a**) EIS profile for different deformations at 0% SOC; (**b**) EIS profile for different deformations at 50% SOC; (**c**) EIS profile for different deformations at 90% SOC; (**d**) EIS profile at different SOC for deformation of 6 mm.

The EIS profile of SIBs under varying degrees of compression deformation are shown in Figure 5a, Figure 5b, and Figure 5c, which illustrate the EIS for SIBs at SOC levels of 0%, 50%, and 90%, respectively, under different compression displacements (i.e., 0–6 mm). Figure 5d shows the EIS characteristics at a compression displacement of 6 mm under different SOC (i.e., SOC is 0%, 50%, and 90%, respectively).

From Figure 5, we can see that the high-frequency region of the EIS profile primarily reflects the ohmic resistance of the battery, including the resistance of the electrolyte, electrode materials, and conductive network. SOC has a minimal effect on ohmic resistance, as observed in Figure 5a–c, since ohmic resistance is mainly determined by material and electrolyte conductivity. However, an increase in SOC and the sodium-ion concentration within electrode materials slightly improves electrolyte ionic conductivity and gradually reducing ohmic resistance (e.g., comparison between Figure 5a,b). However, too high SOC also leads to higher ohmic impedance of SIBs (as shown in Figure 5c), which may be due to the higher SOC leading to high concentration of sodium ions in the electrolyte, which reduces the electrical conductivity, and in turn increases the ohmic impedance of the battery. Additionally, as compression pressure increases, the ohmic resistance of SIBs decreases, as shown in Figure 5b,c. This could result from enhanced physical contact between the electrode and the current collector, improved contact quality, and better electrolyte wettability on electrode surfaces during compression, which collectively reduce interfacial resistance. The mid-frequency region of the EIS profile reflects interfacial impedance, including charge transfer resistance and double-layer capacitance. From Figure 5, we can see that the SOC significantly impacts interfacial impedance. At high SOC levels (i.e., SOC is 90%), the higher sodium-ion concentration in the electrode materials facilitates smoother charge transfer processes, leading to lower interfacial impedance. In addition, high SOC levels increase sodium-ion insertion can also cause electrode swelling and microcracks, which may increase interfacial impedance, as shown in Figure 5d. At low SOC levels (i.e., SOC is 0%), reduced sodium-ion concentration slows charge transfer, increasing interfacial impedance. Under compression, the overall interfacial impedance tends to decrease, as seen in Figure 5a,b. When the compression deformation reaches 6 mm, the interfacial impedance in the mid-frequency region is minimized. This can be attributed to improved electrode–electrolyte interface contact, uniform electrolyte distribution, and promoted interfacial chemical reactions due to compression. The tail of the EIS profile in the low-frequency region reflects Warburg impedance, which indicates the resistance to sodium-ion diffusion within electrode materials. As the electrode becomes saturated with sodium ions, an increase in diffusion impedance is observed, which is a consequence of the limitation of diffusion pathways and the increase in resistance. When the SOC is at 0%, diffusion pathways are less restricted, resulting in lower diffusion impedance, as shown in Figure 5d.

In summary, SOC and compression deformations influence the EIS characteristics of SIBs in distinct ways across different frequency ranges. At higher frequencies, the EIS profile is not significantly affected by the extrusion of the cell, but it can be seen that the impedance of the cell decreases as the extrusion force increases. On the other hand, interfacial impedance, observed in the mid-frequency range, is influenced by both SOC and compression pressure. As SOC increases, the electrode materials undergo more significant expansion and contraction during cycling, which can lead to changes in the interfacial contact between the electrode and electrolyte, thereby altering the interfacial impedance. Furthermore, the compression of the battery under high SOC exacerbates these effects, causing additional interfacial resistance due to the deformation of the electrodes and electrolyte. In the low-frequency region, diffusion impedance is most significantly impacted by the combined effects of high SOC and high compression pressure. The diffusion impedance reflects the ability of ions to move within the electrode materials and electrolyte. At high SOC levels, there is a higher concentration of sodium ions, which increases the internal resistance during ion diffusion. When combined with compression, which further deforms the battery’s internal structure, this leads to a substantial increase in diffusion impedance. The coupling effect of compression and SOC leads to noticeable changes in the entire EIS profile, as shown in Figure 5d, where the shape of the EIS curve shifts under these extreme conditions. Overall, EIS analysis offers valuable insights into the electrochemical behavior of SIBs under different SOC and compression conditions, helping to identify failure mechanisms and improve battery design. By understanding how SOC and compression affect EIS, it is possible to enhance battery performance, reliability, and safety, making this analysis crucial for optimizing battery systems.

### 3.3. Mechanical-Electrical-Thermal Characteristics and Thermal Diffusion Analysis After Compression

#### 3.3.1. Mechanical-Electrical-Thermal Characteristics

**Figure 6:**
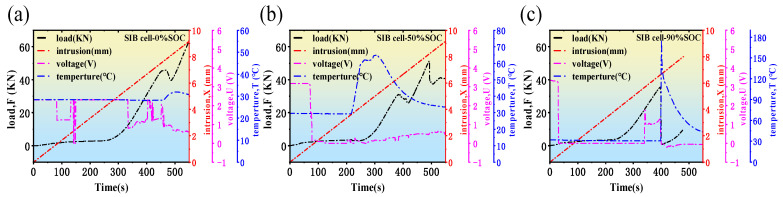
Force–displacement–temperature–voltage relationship of SIB under plane compression load. (**a**) Battery SOC is at 0%; (**b**) battery SOC is at 50%; (**c**) battery SOC is at 90%.

The state of charge of a battery has a direct impact on the distribution of sodium ions within the battery, the degree of material expansion and contraction, and electrode stability. These factors show different behaviors during compression testing. The study in this section evaluates the mechanical, electrical and thermal characteristics of SIBs under SOC of 0%, 50% and 90%. The research results are shown in Figure 6.

When the SOC is 0%, the OCV of the SIB is low (i.e., it is about 2.26 V). This is due to the low sodium-ion concentration in the electrodes, resulting in a low electrode potential. As deformation increases during the test, the applied external force rises gradually, while the OCV remains relatively stable. At 70 s into the test, the OCV drops to around 1.25 V, stays at this level for 40 s, then recovers to 2.26 V. At 300 s, the battery experiences increased stress, reaching a peak at 450 s, accompanied by a rapid temperature rise that peaks at 32 °C at 500 s. When the SOC is at 50%, the OCV is at its nominal voltage of 3.26 V. As deformation increases during the test, the OCV rapidly drops to around 0.3 V at 70 s. After this, the OCV fluctuates around 0.45 V. The temperature reaches a maximum of approximately 65 °C at 300 s, and stress peaks at approximately 350 s. When the SOC is at 90%, the same as at 0% and 50% SOC, the OCV rapidly decreases to around 0.15 V at 40 s under compression. The voltage then recovers to 2.0 V by 345 s, only to drop sharply to 0 V at 400 s. At the same time, the temperature rapidly increases to 179 °C. Moreover, the sample shows a clear explosion sound at around 400 s during compression testing, which is not seen at lower SOC states.

To further clarify the mechanical response of the SIBs during compression, the load–time curves in Figure 6 can be divided into three characteristic stages.

Stage I: Initial accommodation stage. At the beginning of compression (approximately 0–250 s), the intrusion displacement increases continuously, whereas the load remains very low and changes only slightly. This indicates that the external compression mainly causes initial contact deformation of the battery casing and gradual structural accommodation of the internal jelly-roll components. During this stage, the internal structure has not yet developed significant resistance against compression, and both the OCV and surface temperature remain relatively stable.

Stage II: Rapid mechanical loading stage. As compression proceeds further (approximately 250–420 s), the load begins to increase rapidly with time. This behavior suggests that the internal electrode-separator structure becomes progressively compacted, and the battery starts to exhibit stronger mechanical resistance to external deformation. In this stage, pore compression, local electrode deformation, and stress accumulation gradually intensify. Meanwhile, the electrical response becomes more sensitive, especially at medium and high SOC, where obvious voltage drops and temperature increases begin to appear.

Stage III: Failure stage. When the compression reaches a critical level (after approximately 420 s, depending on SOC), the battery enters the failure stage. In this stage, the load reaches its peak value or shows obvious fluctuation, while the OCV drops sharply and the temperature rises rapidly. These changes indicate the occurrence of severe internal damage, such as separator rupture, electrode contact, internal short circuit, and structural collapse. In particular, under 90% SOC, the temperature rises dramatically and an explosion-like sound is observed, indicating a much more violent failure process.

These results demonstrate that the mechanical response of the battery under compression is not characterized by a simple monotonic load increase, but rather by a transition from initial structural accommodation to rapid load-bearing and finally to catastrophic failure. This staged evolution provides important evidence for understanding the coupling relationship between mechanical deformation and the electrical and thermal responses of SIBs.

**Figure 7:**
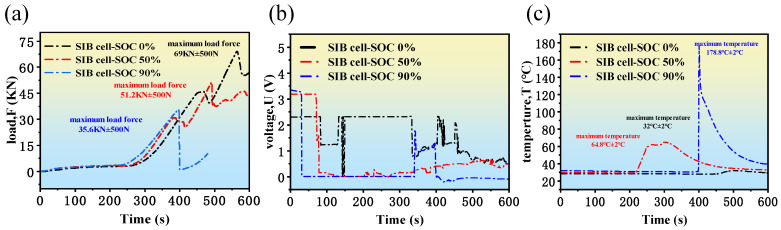
Comparison of forces, voltages and temperatures at different SOCs. (**a**) Squeeze stress comparison at different SOCs; (**b**) OCV comparison at different SOCs; (**c**) temperature comparison at different SOCs.

The beforementioned research results indicate that the mechanical stress, OCV, and surface temperature responses of SIBs differ significantly under varying SOC and compression deformation conditions. To provide a clearer comparative analysis, the mechanical stress, OCV, and surface temperature of SIBs at different SOCs during compression are shown in Figure 7.

In Figure 7a, it can be observed that when the SOC is 0%, the concentration of sodium ions in the electrode material is low, and the volume change in the material is minimal. Therefore, it exhibits better structural stability. When the SOC is 50%, the concentration of sodium ions in the electrode material is at a moderate level. In this case, the increase in stress is reflected in localized microcracks and microscopic damage. The first yield point corresponds to a stress that is significantly lower than that at SOC = 0%, but it does not cause significant damage to the overall structural stability, indicating that the battery has certain structural endurance under moderate deformation. When the SOC is 90%, the concentration of sodium ions approaches saturation in the electrode materials, and the electrode lattice undergoes significant expansion during sodium-ion insertion. This volumetric expansion is particularly evident in layered oxide cathodes such as NaCrO_2_, where sodium intercalation causes lattice expansion along the interlayer direction. Previous studies have reported that the volume expansion of layered sodium transition metal oxides during sodiation can reach approximately 5–10% [31], which significantly alters the mechanical stability of the electrode structure.

Under high SOC conditions, the electrode materials are already in an expanded state due to sodium-ion insertion. When external compression is applied, the available space for further deformation becomes limited, resulting in a rapid accumulation of internal mechanical stress within the battery structure.

In addition to volume expansion, the distribution of sodium-ion concentration within the electrode and electrolyte also plays a crucial role in stress evolution. At high SOC, the sodium-ion concentration gradient between the electrode surface and the electrolyte becomes more pronounced. During compression deformation, the porous electrode structure may experience pore collapse and electrolyte redistribution, which restricts ion transport pathways and leads to localized ion accumulation within certain regions of the electrode [14].

This non-uniform ion distribution can generate localized electrochemical reactions and induce diffusion-induced stress inside the electrode particles. The relationship between ion concentration variation and stress evolution can be expressed as:

(1) $$\sigma =\frac{E\beta \Delta c}{1-\nu }$$

where E represents the elastic modulus of the electrode material, β is the partial molar volume, Δc denotes the variation in sodium-ion concentration, and ν is Poisson’s ratio. According to this relationship, larger ion concentration variations at high SOCs can significantly increase internal stress during mechanical deformation.

Therefore, under high SOC conditions, the combined effects of electrode volume expansion and non-uniform ion concentration distribution lead to faster stress accumulation in the battery structure. During deformation, the rate of internal stress increase in the SIBs is significantly faster than that at SOC = 0% and SOC = 50%. The local stress may exceed the yield strength of the material, causing crack propagation, particle shedding, and structural damage, resulting in a noticeable explosion sound during compression.

Regarding the influence of different SOC values on OCV during the compression process, as shown in Figure 7b, when the SOC is 0%, the concentration of sodium ions in the electrode material is low, and the OCV is generally low. The deformation has relatively little impact on the OCV (for example, when SOC = 0%, the first voltage drop occurs around 70 s, with a voltage change from 2.25 V to 1.25 V, a change of 1 V; while for SOC = 50% and SOC = 90%, the first OCV drop occurs at 68 s and 40 s, with changes of 3.02 V and 3.21 V, respectively). This is because the electrode structure at low SOC is relatively stable, and internal stress is low, making it less affected by mechanical deformation. When the SOC is 90%, the sodium ion concentration is close to saturation, and the electrode material is more prone to structural damage or microcracks under stress. Mechanical deformation may cause additional microstress in the electrode material, leading to the failure of active materials inside the battery and side reactions, which further affects the stability of the OCV. Under deformation conditions, the OCV fluctuations are most noticeable at SOC = 90%, indicating that the structure of high SOC batteries is relatively the least stable under mechanical stress.

In Figure 7c, the surface temperature of the SIBs rises due to internal short circuits, increased impedance, and stress release in the electrode material. Different SOC states have a significant impact on the amplitude of temperature changes and the rate of temperature rise. When the SOC is 0%, the internal activity of the SIB is low, and the friction and heat accumulation caused by mechanical deformation are minimal, so the surface temperature does not change significantly. When the SOC is 50%, sodium ions migrate more actively within the SIB, and stress release occurs after the electrode material is stressed. When the SOC is 90%, due to the sodium ion concentration approaching the upper limit of the electrode material, mechanical deformation is more likely to cause microscopic structural damage, leading to internal short circuits and rapid accumulation of internal energy. At the same time, internal side reactions in the battery are more active at high SOC, and the heat released from these reactions further exacerbates the surface temperature rise, which may even lead to an explosion.

In summary, the mechanical, electrical, and thermal characteristics of SIBs under compression vary significantly with SOC. At low SOC, the OCV remains stable despite increasing deformation, with only a slight rise in temperature. As the SOC increases to a moderate level, the OCV decreases rapidly, and the temperature rises more substantially, indicating higher internal stress and greater material deformation. At high SOC, the OCV initially drops sharply under compression, followed by a partial recovery, but eventually decreases further as the battery undergoes significant structural damage. Concurrently, the temperature increases dramatically, and the battery may emit explosion-like sounds, suggesting severe internal failure. These findings highlight the critical influence of SOC on the electrochemical, mechanical, and thermal behaviors of SIBs under mechanical stress, with high SOC states exhibiting greater vulnerability to failure. The reason for these phenomena is relative to complex reactions within the LIBs during tests; for example, the dynamic evolution mechanism of internal short circuit (ISC) caused by compression lead to thermal runaway and other side reactions. However, with the advancement of material technology, the application of low-temperature materials in SIBs will further enhance the comprehensive performance of SIBs.

#### 3.3.2. Battery Thermal Runaway

**Figure 8:**
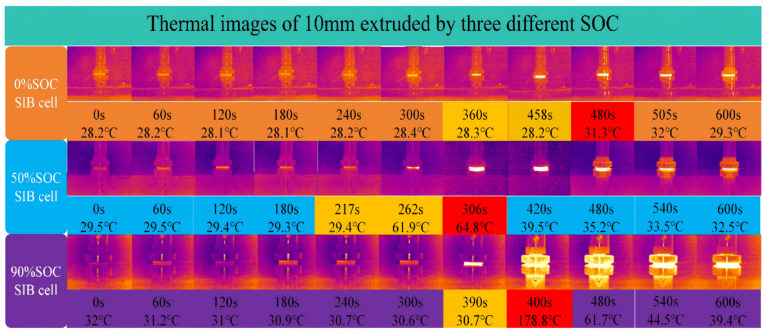
Thermal diffusion of SIBs during compression.

The thermal runaway diffusion characteristics and thermal imaging of the battery at different SOCs during the compression deformations are shown in Figure 8.

In Figure 8, at the initial stage, the temperature of the SIBs under different SOC conditions is approximately 28–32 °C. As the compression test progresses, the temperature remains relatively stable for the first 30–50 s, then increases at rates that depend on SOC. For SOC = 0%, the onset of rapid temperature rise occurs at approximately 65 °C, with a maximum surface temperature of 78 °C and an average heating rate of 0.3 °C/s. The low sodium-ion concentration and minimal electrochemical activity limit heat generation, resulting in small local temperature increases around the compression area and high thermal stability. For SOC = 50%, the onset of rapid heating occurs at ~105 °C, with a peak surface temperature of 165 °C and an average heating rate of 1.2 °C/s. Moderate sodium-ion concentration and increased internal impedance promote local heating during compression, leading to more pronounced thermal gradients compared to SOC = 0%. For SOC = 90%, rapid heating begins as early as 95 °C, with the surface temperature peaking at 179 °C and an average heating rate of 4.2 °C/s. High sodium-ion concentration and maximal electrode expansion produce significant heat, which accumulates faster than it can diffuse, occasionally resulting in minor explosions and large localized thermal gradients, indicating poor thermal management and a high risk of thermal runaway.

Overall, while the initial temperatures are similar across SOCs, the onset temperature, maximum surface temperature, and heating rate all increase with SOC, reflecting the stronger coupling between electrochemical activity, mechanical compression, and thermal response at higher states of charge. These quantitative metrics provide a clear basis for evaluating the thermal stability and safety of SIBs under mechanical stress.

### 3.4. Morphological and Crystal Phase Characteristics

**Figure 9:**
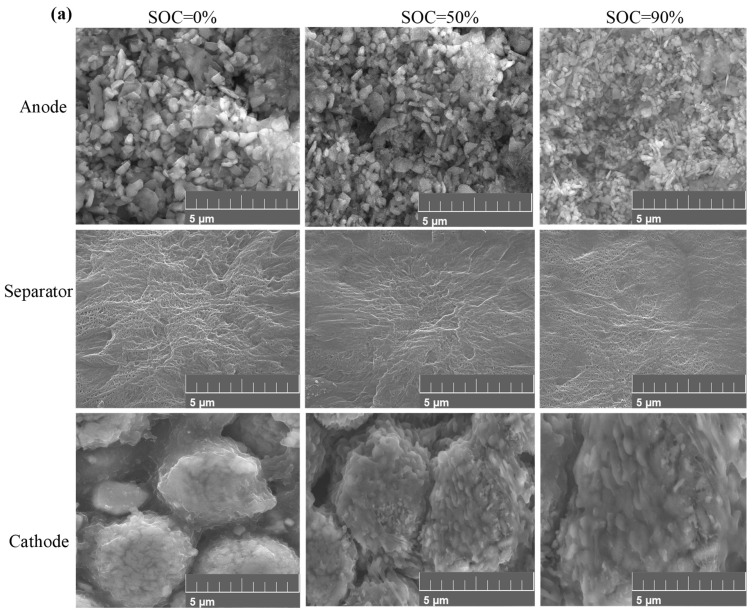

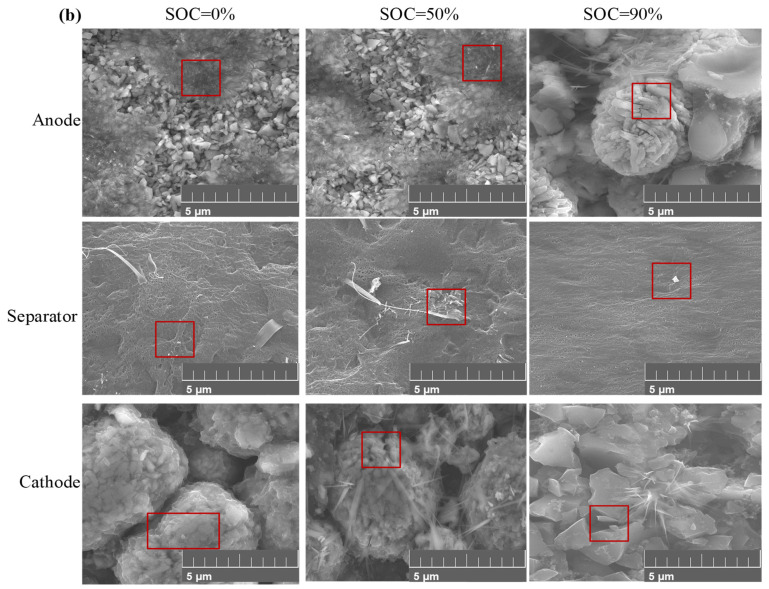
Morphological characteristics under SEM. (**a**) Normal morphological features; (**b**) morphological features after compression failure.

To further explore the degeneration in the morphology of the positive electrode, negative electrode, and separator of the SIBs after compression failure, we used scanning electron microscopy (SEM) and X-ray diffraction (XRD) to analysis the morphology and crystal phase structure. The results are shown in Figure 8 and Figure 9.

In Figure 9, it is evident that after compression, the positive electrode material of the SIBs exhibits visible cracks and particle peeling on the surface of certain particles, which occur due to the external force applied during compression. These physical changes, including cracks and particle fragmentation, have a direct impact on ion transport and conductivity. Cracks disrupt the continuity of the electrode material, creating pathways that hinder efficient ion movement between the electrolyte and the electrode surface. Particle peeling further worsens this effect by exposing the inner, less conductive regions of the material, reducing the overall active surface area available for ion exchange. The appearance of a thin layer on the surface of some regions (shown in Figure 9b) is likely a result of side reactions between the electrolyte and the electrode material, further impeding ion transport and lowering overall conductivity. Similarly, the compression-induced changes in the separator, where micropores become compressed and some pores become irregular, significantly affect ion flow. The damage to the separator, including tearing and the formation of wrinkles, reduces its mechanical strength and flexibility. This degradation increases the risk of internal short circuits, which could further compromise the overall battery performance. In the negative electrode, the noticeable particle fragmentation and deformation lead to a decrease in porosity, causing the particles to become more tightly packed. While this may seem to improve structural integrity, it actually restricts ion movement, as the compacted structure inhibits the free flow of sodium ions, further reducing conductivity. These combined effects of cracks, particle fragmentation, and separator damage highlight the critical role that mechanical deformation plays in reducing ion transport efficiency and overall conductivity in SIBs. Comparing the morphology of the positive electrode, separator, and negative electrode before and after compression at different SOCs, we find that the morphology changes are not significant before and after compression. This could be because the battery is in a discharged state due to short-circuiting, leading to final energy release.

**Figure 10:**
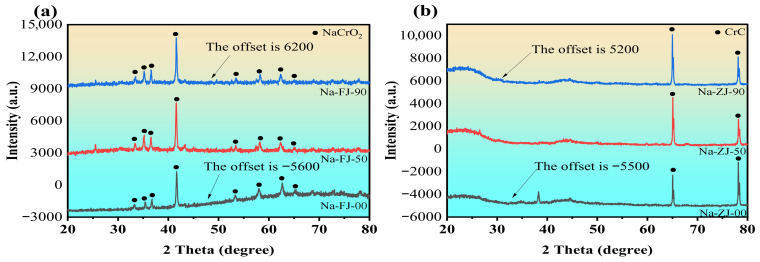
XRD patterns of the cathode and anode material. (**a**) XRD patterns of the anode material; (**b**) XRD patterns of the cathode material.

In this paper, the positive electrode material of the SIBs sample uses a sodium-based layered oxide (i.e., NaCrO_2_), which undergoes electrochemical reactions by the intercalation and deintercalation of sodium ions during charge and discharge. The negative electrode materials are hard, carbon-based materials (CrC), which have disordered carbon layer stacking structures. These compounds exhibit specific diffraction peaks in XRD patterns, as shown in Figure 10. In Figure 10a, the XRD curves for each state (i.e., SOC is 0%, 50%, and 90%) after compression show clear, sharp diffraction peaks, with a high diffraction peak at 42° that is stable in position, indicating that the crystal structure of the positive electrode material remains intact and the interplanar spacing is stable during this experiment. Figure 10b shows the XRD patterns of the negative electrode after compression at SOC values of 0%, 50%, and 90%. The XRD pattern of the compressed hard carbon negative electrode exhibits typical CrC diffraction peaks, especially a significant strong peak at 65°, which is one of the characteristic peaks of CrC. Comparing the XRD patterns of the negative electrode at different SOC values, no noticeable changes in diffraction peak position, width, or intensity occur, indicating that different SOC values in this experiment do not lead to deformation of the crystal phase structure. The crystal structure, grain boundary stability, and crystallinity of the material remain stable.

### 3.5. Discussion on Practical Applications

The findings of this study have important implications for the practical application of SIBs, particularly in electric vehicle safety and industrial energy storage. Owing to the abundance and relatively low cost of sodium, SIBs are widely regarded as a promising alternative to lithium-ion batteries; however, their mechanical reliability and electrochemical stability under abusive loading conditions remain critical issues for large-scale deployment. The present results show that the mechanical and thermal responses of SIBs are strongly dependent on the state of charge (SOC), with highly charged cells exhibiting more severe deformation-induced electrochemical degradation, greater thermal instability, and a higher risk of hazardous failure. These behaviors may be associated with the coupled effects of mechanical compression, ion redistribution, electrode structural damage, and diffusion-induced stress. During compression, non-uniform deformation can disturb Na-ion transport pathways and generate local concentration gradients within the electrodes. Such gradients may give rise to diffusion-induced stress, which can further promote particle cracking, interfacial degradation, loss of active material, and localized heat generation. Although the present study does not provide a fully quantitative stress model because spatially resolved Na-ion concentration, diffusion coefficients, and local mechanical parameters were not directly measured, the concept of diffusion-induced stress provides a useful mechanistic framework for interpreting the interaction between ion transport and material degradation under compression. In electric vehicle applications, the SOC-dependent failure characteristics of sodium-ion batteries (SIBs) are particularly relevant to crash scenarios, impact events, and other forms of mechanical abuse, where cells may experience severe local deformation. The measured mechanical stress, open-circuit voltage (OCV), and temperature evolution can be interpreted through the lens of diffusion-induced stresses within the electrodes, where localized compression generates concentration gradients that induce internal stress, promoting particle cracking, interfacial degradation, and localized heat generation. Specifically, at low SOC (0%), the electrodes contain few sodium ions, leading to minimal volumetric expansion, low internal stress, and limited microstructural damage, which corresponds to relatively stable OCV and a modest temperature rise. At moderate SOC (50%), the electrode sodium concentration is higher, and mechanical compression produces localized microcracks and small structural deformations, causing noticeable OCV fluctuations and moderate heating. At high SOC (90%), the electrodes are nearly saturated with sodium, and external compression rapidly accumulates internal stress, resulting in extensive particle cracking, interfacial degradation, accelerated side reactions, sharp OCV drops, rapid surface temperature rise, and, in extreme cases, explosive failure. These observations illustrate the strongest coupling between mechanical deformation, diffusion-induced stress, and electrochemical–thermal responses under high SOC conditions. It should be noted that the stress model applied in this study is based on linear elastic theory and assumes homogeneous, isotropic electrode materials. While this provides a first-order mechanistic framework to interpret diffusion-induced stress and its interaction with deformation, it does not capture plastic deformation, fracture, or heterogeneous electrode structures observed experimentally. Therefore, the model is most valid under conditions of small elastic strains and relatively uniform material behavior. Under high SOC and severe compression, where significant plasticity, cracking, and inhomogeneous deformation occur, the model serves as a qualitative tool to rationalize trends rather than a quantitative predictor of local stress distributions. Therefore, managing high-SOC exposure during high-risk events, enhancing cell-level mechanical protection, and integrating deformation-sensitive safety strategies into battery management systems are essential for reducing the probability of thermal runaway. For grid-scale and industrial energy storage applications, understanding how diffusion-induced stresses correlate with mechanical load evolution and electrochemical signals can inform safer module design, improve fault diagnosis, and optimize operational lifetime. Overall, these findings highlight the critical importance of considering the interplay between SOC, mechanical deformation, diffusion-induced stress, and electrochemical–thermal evolution to ensure the safe and reliable deployment of SIBs.

In conclusion, while SIBs show great promise for both electric vehicle and industrial energy storage applications, addressing the safety concerns highlighted in this study is essential for their successful deployment. Future research should focus on improving the structural integrity and thermal management of SIBs to ensure their safe and efficient operation in real-world scenarios.

## 4. Conclusions

In this study, we performed a comprehensive investigation into the failure mechanisms, morphology, mechanical, electrical, and thermal characteristics of SIBs under different deformation conditions. The research findings provide critical insights into the electrochemical performance, output behavior, and structural optimization of SIBs for practical engineering applications. The following conclusions can be drawn from the results:

1. Different frequency bands of EIS are affected by SOC and squeezing deformation to varying extents. Ohmic impedance is primarily influenced by deformation, while surface impedance is impacted by both SOC and deformation. The diffusion impedance at low frequencies increases most significantly under high SOC and high squeezing force conditions.
2. SOC significantly affects the OCV, surface temperature, and mechanical stress during compression tests. High SOC makes OCV more susceptible to fluctuations and increases the likelihood of thermal runaway. Quantification shows that internal stress rises rapidly at high SOC, making the battery more prone to structural damage. In contrast, at low and moderate SOC, stress increases more gradually, resulting in less structural damage. This highlights the critical importance of SOC in managing battery performance under mechanical stress.
3. Significant differences were observed in the thermal runaway characteristics depending on SOC. At low SOC, thermal runaway is characterized by a slow temperature increase and uniform distribution. However, at high SOC, the runaway is more rapid, concentrated, and accompanied by a steep temperature gradient, indicating a higher risk of failure and safety hazards.
4. After extrusion, significant morphological changes were observed in the positive and negative electrodes and separators of SIBs. The compression process leads to particle fragmentation, reduction in porosity, and crack formation in the electrode materials, which disrupts ion channels and conductive paths, thereby degrading battery performance. The diaphragm’s microporous structure is also compressed, increasing the risk of short circuits.

The findings from this study can be applied to the optimization of SIBs in practical engineering contexts, particularly in energy storage systems and electric vehicles. The insights into the effects of SOC on battery performance under deformation can guide the design of more robust batteries that are better equipped to handle mechanical stress and high-temperature environments. Additionally, future work should focus on comparative studies between sodium-ion and lithium-ion batteries, especially in the context of lithium iron phosphate (LFP) and ternary lithium-ion batteries, to better understand their respective failure mechanisms and enhance their safety and performance under similar deformation conditions.

## Figures and Tables

**Table 1:** Parameters of SIBs.

Item	Parameters
Charging/Discharging Capacity ± 0.1 Ah	1.25
AC Internal Resistance (mΩ) ± 0.2 mΩ	≤1.6
Cell Weight (g) ± 0.5 g	40.1
Operating Voltage Range	2.0–4.2
Charging Temperature Range (°C)	0–45
Discharge Temperature Range (°C)	−20–50
Continuous Charging/Discharging Current	1.5 C
Nominal Voltage(V) ± 0.1 V	3.1

**Table 2:** Equipment models and specifications.

Specifications	Units	Values
Charging/Discharging equipment		
Measuring range of voltage	V	0–10
Measuring range of currents	A	0~20
Sample rate	Hz	1
Response time	mS	10
Potential increments	mV	0.1
Adjustable temperature range of temperature chamber	°C	−40–80
Temperature rise rate	°C/min	0.1
Volume of temperature chamber	L	60
Electrochemical workstation		
Measuring range of voltage	V	−10–10
Max. continuous current	mA	250
Bandwidths	MHz	1
Potential increments	mV	0.1
Minimum sample interval	µs	1
Bias current	pA	≤10
Maximum sampling rate	MHz	1
Update rate	MHz	10
Max. data length	K	16,384
Accuracy of added potential	mV	±1
ACV frequency range	kHz	0.1–10

## Data Availability

The original contributions presented in this study are included in the article. Further inquiries can be directed to the corresponding author(s).

## Nomenclature

SIBs	Sodium-ion batteries
EVs	Electric vehicles
SOH	State of health
EIS	Electrochemical impedance spectroscopy
AC	Alternating Current
XRD	X-ray diffraction
OCV	Open-circuit voltage
$R_{sei}$	Internal resistance of the SEI
SOE	State of energy
$R_{ct}$	Internal resistance of charge transfer
LIBs	Lithium-ion batteries
SOC	State of charge
SEM	Scanning electron microscopy
ECM	Equivalent circuit model
RC	Resistance-capacitance
SEI	Solid electrolyte interface
$R_{\Omega }$	Ohmic internal resistance
$Q_{sei}$	Constant phase angle element
NMC	LiNi_x_Mn_z_Co_y_O_2_
W	Warburg impedance
